# Occurrence of Tetracycline Residue in Table Eggs and Genotoxic Effects of Raw and Heated Contaminated Egg Yolks on Hepatic Cells

**DOI:** 10.18502/ijph.v49i7.3590

**Published:** 2020-07

**Authors:** Abbas KAMALI, Maryam MIRLOHI, Mahmod ETEBARI, Sohila SEPAHI

**Affiliations:** 1. Food Security Research Center, Department of Food Hygiene and Safety, Qazvin University of Medical Sciences, Qazvin, Iran; 2. Food Security Research Center, Department of Food Science and Technology, School of Nutrition and Food Science, Isfahan University of Medical Sciences, Isfahan, Iran; 3. Department of Pharmacology, Isfahan Pharmaceutical Sciences Research Center, School of Pharmacy and Pharmaceutical Sciences, Isfahan University of Medical Sciences, Isfahan, Iran

**Keywords:** Tetracycline residues, Egg, Boiling, Comet assay

## Abstract

**Background:**

This study measured tetracycline residue in table eggs sampled from Isfahan markets in 2015 and assessed the toxic effects of heated egg yolk on hepatic cells

**Methods:**

Forty commercial and six locally produced eggs were randomly collected and tested for tetracycline resides using competitive ELISA with a mean recovery value of 65.22% and limit of detection (LOD) of 4 ng/g. For the seven most contaminated samples, albumen and yolk were examined individually and, despite the very low detected level in the yolk, the samples were subjected to boiling for 10 min. Finally, the DNA damaging properties of the raw or treated egg yolks on hepatic cells were investigated using the comet assay.

**Results:**

The residue levels ranged from <LOD to 9.77 ng/g with a mean value of 4.95 ng/g, which was far below the maximum residue level (MRL) of tetracycline in eggs (200 ng/g) established by the EFSA. The concentration of tetracycline residue in the albumin and yolk of the seven most contaminated samples was 4.75 ng/g and 6.83 ng/g, respectively, while boiling reduced it by 20%. Heat treatment induces DNA damage in HepG2 cells. Heating resulted in a marked increase in the comet length, % DNA in the tail, and tail moment parameters by 60%, 3000%, and 5000%, respectively.

**Conclusion:**

Despite the low concentration of tetracycline residue in samples, heat treatment can create degenerative compounds from tetracycline that can cause DNA damage in an in vitro model.

## Introduction

Tetracyclines are a group of antibiotics derived from *Streptomyces* spp*.*, with broad-spectrum activity against Gram-positive and -negative aerobic and anaerobic bacteria (1) with protein–synthesis- inhibition as their major mechanism of antibacterial activity (2). Tetracycline antibiotics are commonly used in chicken warehouses for disease prevention, medication (3), and egg weight enhancement effects (4). Regarding the various poultry production practices, few medications are authorized for administration to laying hens, which reduces the likelihood of exposure to antibiotics and the risk of contamination with antibiotic residues in eggs. However, antibiotics may impose a weight-enhancing effect on chicken eggs, which can be an incentive to use antibiotics, such as as tetracycline, in laying hens (5).

Like any other veterinary drug, the inappropriate and haphazard use of tetracycline, as well as improper withdrawal periods in poultry production practice, may lead to tetracycline residue deposition in eggs (6). The presence of tetracycline residue (TCs) in eggs has been reported by both independent studies and governmental food safety monitoring programs.

As they have a biological value of ∼100, eggs are an excellent source of high quality, readily utilizable protein in the human diet and are used as a reference protein when assessing the bioavailability of proteins. Lipid in eggs is comprised of essential components, such as lecithin and choline, which function as a component of neurotransmitters and are particularly effective in the development of the nervous system and brain. Choline also improves cognitive and learning performance in infants and aids memory in the elderly (7).

Considering high nutritional values of eggs, it is not surprising that eggs area major component of the recommended early solid feeding regimen for infants. Moreover, the yolk is considered to be a valuable ingredient to be added to infant formula with a nutritional quality similar to that of breast milk (8) .

The occurrence of TC residues in eggs along with a rise in its consumption could lead to elevated intake of TC derivatives with the associated risk of its potential hazards (9). Notably, because TCs residues in food are known to pose hazards to public health, especially in children, some of the associated adverse effects are more crucial, including hypersensitivity reactions and teeth yellowing

In the first semester of pregnancy, exposure to TCE has been associated with mutagenic and teratogenic effects in fetus. Additionally, renal and immune disorders, as well as toxic effects, have been linked with prolonged tetracycline in-take (10).

In the presence of nitrite compounds, tetracycline may enhance the formation of nitrosamine with a subsequent increase in carcinogenicity and allergic reactions. Moreover, prolonged exposure to oxytetracycline residue through food intake has been linked to the development of bacterial resistance and changes in intestinal microflora (11).

The joint FAO/WHO Expert Committee on Food Additives (JECFA), at its 50 ^th^ Meeting in 1998, established an acceptable daily intake (ADI) of 0–0.03mg/kg body weight for the tetracycline compounds (oxytetracycline, tetracycline, and chlortetracycline) alone or in combination (12). Tetracyclines are heat liable and food studies on milk, chicken, and egg showed that heating can decrease tetracycline residues (13,14). Recently, some studies have investigated whether antibiotic residues and their degraded compounds are genotoxic. Studies of the effects of high heat temperatures on antibiotic residues indicated that the antibacterial effects of tetracyclines declined, while their toxicity increased after heating. This effect was accompanied by the formation of new compounds in the heat-treated samples, likely indicating the formation of new tetracycline epimers (15).

To date, very few studies have been carried out on this subject. This present study considers the genotoxic effects of tetracycline residue in egg yolk at a concentration that are commonly found in eggs sold in the markets of Isfahan City, Iran. Herein, we investigated whether or not the weight of eggs, yolks, albumen, crusts, or albumen/yolk weight ratio have any association with the tetracycline residue concentrations in egg samples. Additionally, we compared industrially produced samples and the domestic ones to detect potential differences in tetracycline residue concentrations.

## Materials and Methods

This study was carried out in four sequential steps. First, the occurrence of tetracycline residues in eggs sold at the market was investigated. Second, the seven most contaminated samples were chosen and the concentration of residues was determined in both albumen and yolk separately. Third, egg yolks were cooked and changes in the level of contamination were studied. Finally, the genotoxic effects of cooked egg yolks were investigated using a comet test.

A tetracycline ELSA commercial kit, including tetracycline standards (LOT: ON6857 and LOT: ON6859), dilution buffer (LOT:ON6837), rinsing buffer (LOT:PN5343), stop solution (LOT:PN5181), substrate/chromogen (tetramethylbenzid, TMB; LOT:PN5051), and tetracycline-HRP (conjugate; LOT:ON7129) were provided by Europroxim. Ethanol was purchased from Anahita (Iran), methanol(100%) was purchased from pars chemic (Iran), and cell culture reagents (LOT: ON6855) were purchased from Europroxima.

Normal melting point agarose (NMA) was purchased from SinnagenCo. (Iran). Hep G2 cells were purchased from the Pasture Institute (Iran). RPMI-1640, FBS, and antibiotics were purchased from PAA Co. (Australia). Low melting point agarose (LMA), NaHPO, Ethidium bromide, and KCL were purchased from Sigma Co. (USA). EDTA, HO, Ethanol, NaCl, NaOH, Tris, and Triton X-100 purchased from Merck Co.(Germany).

### Sample preparation, tetracycline extraction, and measurements

Overall, 40 egg samples from 13 commercial and six local farms and were tested for the prevalence of tetracycline residue during the year 2105. Samples were selected using a simple random sampling method. Eggs were carefully weighed before the experiment, cleaned using an ethanol-soaked cloth, and gently cut in the middle using a cleaned sharp knife. Albumen and yolk were separated and each was weighed in a separate clean container; the ratio of albumin-to-yolk was determined for each sample. The separated parts were distributed in vials, covered tightly, and stored in a freezer (−70 °C) for storage until subsequent experiments. At the time of the experiment, defrosted yolk and albumen from each sample were mixed well together at their original ratio in a micro-tube, from which 1 g was taken for the following experiment.

The sampling procedure allowed us to track the residue in the contaminated samples, either in the yolk or albumen. Extraction and measurements of tetracycline were performed according to competitive enzyme immunoassay protocol for screening and quantitative analysis of tetracycline using commercial kit (Eruproxia, Hungry). A mean recovery value of 65.22% and limit of detection (LOD) of 4 ng/g were obtained for the applied test. For tetracycline measurements in the yolk or albumen, 1 g from each component was tested as the sample. To measure the effects of heat on tetracycline residues, the yolk of the seven most contaminated samples was heated for 10min at 100 °C in a water bath, cooled to room temperature, and re-examined to test the remaining tetracycline residue level.

### Comet test

Egg yolk samples with the highest tetracycline concentration were used for comet testing the comet assay so that the raw and heat-treated types with and without standard addition were examined for comet length, % DNA tail, and tail moment.

### Materials for the comet Assay

Normal melting point agarose (NMA) was purchased from CinnagenCo. (Iran). HepG2 cells were purchased from the Pasture Institute (Iran). RPMI-1640, FBS, and antibiotics were purchased from PAA Co.(Australia). Low melting point aga-rose (LMA), NaHPO, Ethidium bromide, and KCL were purchased from Sigma Co.(USA). EDTA, HO, Ethanol, NaCl, NaOH, Tris, and Triton X-100 were purchased from Merck Co.(Germany).

### Cell culture

RPMI medium that contained 7% fetal bovine serum and 1% penicillin/streptomycin, to avoid from growth of undesirable and pathogenic bacteria, was used to cultivate the human hematoma (HepG2) cell line. This cell line was incubated under 5% CO _2_ at 37 °C in a microfilter plate. Cells were incubated with extracted samples (200μl) of raw or heated yolk with 800μl PBS. The upper-medium of wells was thrown away and washed with PBS. After trypsinization, 1 ml medium was added to each Falcon tube to use for the next stages of the comet assay.

### Alkaline comet assay

Overall, 300μl cell suspension (10 ^6^ cells/ml) was mixed with 2ml 1%LMA and these solutions were placed on percolated slides with 1% NMA and then covered by cover glasses for 8 min. For extracted nuclear DNA, slides were incubated with lysis solution (pH=10). The lysing process was achieved in a dark room at 4 °C for 40 min and then rinsed with distilled water for 20 min to remove excess lysis solution. After this stage, slides were incubated with electrophoresis buffer (pH>13) for 40 min with 25V and 300MA. In the next step, they were rinsed with distilled water to remove excess alkaline buffer and then incubated in neutralization solution (Tris 0.4 M, pH>7.5) for 10 min. Slides were covered with sufficient dye solution (20 μg/ml ethidium bromide) for 5 min and then washed with distilled water. Finally, comets were visualized under ×400 magnification using fluorescence microscopy with an excitation filter of 510–560 nm and a barrier filter of 590 nm. All stages of the comet assay were performed at 4 °C in dark conditions and all solutions were prepared freshly and used cool.

### Statistical analysis

All statistical analyses were performed using SPSS ver. 18 (Chicago, IL, USA). Data for tetracycline residues in egg samples were presented as means of the results obtained from several samples taken from each brand. Pearson coloration tests at 0.01 and 0.05 levels were performed to investigate the existence of a correlation between tetracycline residues and certain physical characteristics of the samples. Additionally, one sample T-test and ANOVA analysis were applied for mean value comparisons. *P-*values less than 0.05 were considered to be statistically significant.

## Results

### Tetracycline residue measurements

Table 1 shows the results of tetracycline measurement in the tested samples. All of the examined eggs were positive for tetracycline residue. With an average value of 3.10ng/g, minimum and maximum levels of tetracycline were0.56 and 9.77ng/g, respectively. Considering the regulated level by EFSA of 200ng/g, all samples were considered to be acceptable.

**Table 1:** Results of tetracycline measurements in eggs by brand

*** Brands ***	N ^**^	Mean Concentration ±SD ^*^ (ng/g)
A	5	4.515±0.83
B	3	11.036±0.27
C	3	10.364±1.47
D	3	4.550±0.53
E	3	4.937±1.43
F	3	4.282±1.07
G	3	4.413±0.109
H	3	5.237±0.80
I	2	4.371±1.00
J	3	4.026±0.88
K	3	5.060±0.33
L	3	<LOD
M	3	4.541±0.16
Local	6	<LOD

* Average values of measured tetracycline concentration ± SD in samples of each brand.

N^**^ The number of tested samples from each brand.

Pearson correlation test results showed no significant correlation at the 0.05 level between tetracycline residue concentrations and any of the parameters tested of physical characteristics, including egg weight, albumen weight, yolk weight, crust weight, and albumen-to-yolk ratio; however, a significant correlation at the 0.01 level was detected between these parameters.

A comparison of tetracycline residues in the samples produced in the industrial warehouses and domestic products showed that although both were detected in an acceptable range, the average amount of analytics in the industrially produced eggs (5.14±2.16ng/g) was relatively higher than that in the domestic samples (3.62±0.73ng/g).

A comparison of the tetracycline residue levels in albumen, yolk, and heat-treated yolk of the seven most contaminated samples is presented in Table 2. Comparison of the means showed significantly higher concentrations of tetracycline in yolk (6.83±1.03ng/g) than in albumen (4.75±0.75ng/g; *P*<0.05). Heating the most contaminated egg yolk samples led to an ∼20% reduction in TC residue to 5.85±1.24 ng/g, which was significantly different from the relevant concentration in raw yolk samples (*P*>0.05). Results of the comet assay indices, including comet length, % DNA in the tail, and tail moment, are presented in Table 3.

**Table 2:** Tetracycline concentrations (ng/g) in egg yolk and albumen among the seven most contaminated samples

*** Sample No. ***	*** 1 ***	*** 2 ***	*** 3 ***	*** 4 ***	*** 5 ***	*** 6 ***	*** 7 ***
Albumen	4.6	4.7	4.4	4.7	5.4	4.5	4.6
Yolk	6.9	6.4	7	6.7	6.5	6.5	7.8
Heated Yolk	5.4	5.9	5.9	6.2	5.2	5.7	6.2

Each data presents an average of two repeated measurements

**Table 3:** Comet test results for raw and heat-treated yolk samples with and without the addition of standard^*^

*** Variable ***	*** Comet length (mean±SD) ***	*** %DAN in tail (mean±SD) ***	*** Tail moment (mean±SD) ***
Negative control	50.119±6.2	0.17±0.08	0.006±0.0027
Raw yolk ^b^	89.411±0.51	0.42±1.11	0.16±0.26
Spiked raw yolk ^c^	105.637±2.99	2.21±4.34	1.031±2.67
Heat treated yolk ^d^	143.216±0.64	14.312±1.79	9.402±1.18
Heat treated spiked yolk ^ e ^	206.483±4.47	24.367±3.12	31.042±6.29

* Data are presented as average values of 99 repeated measurements.

a: Raw yolk samples with the highest concentration of tetracycline,

b: a+100 ng tetracycline,

c and d: heat treated types of a and b, respectively

**Fig. 1:**
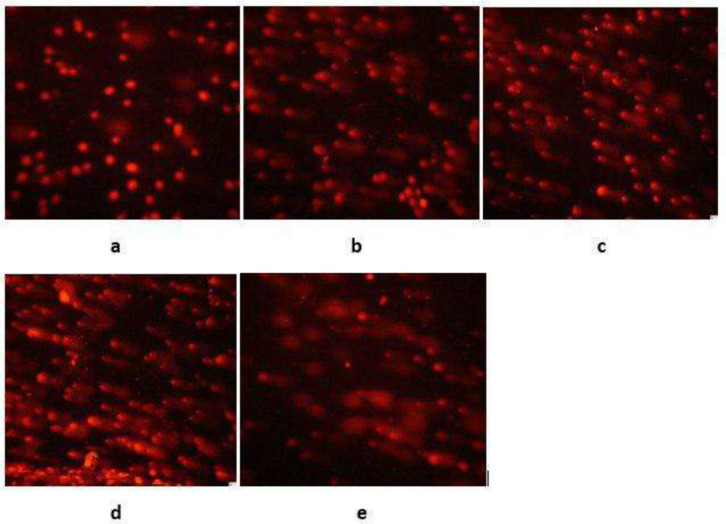
Images of the comet test obtained using a fluoresce microscope.a: Negative control, b: Raw yolk, c: Heat-treated yolk, d: Raw yolk + 100ng tetracycline e: Heat treated yolk+ 100ng tetracycline

Shown in Fig. 1, the comet test indicates the apparent effect of heating on the genotoxicity criterion; heating resulted in a marked increase in comet length, % DNA in tail, and tail moment parameters by 60%, 3000%, and 5000%, respectively. A similar trend was found for samples for which a standard solution of tetracycline was added; comet length, % DNA, and tail moment parameters were increased 96%, 1100%, and 3000%, respectively. Nevertheless, the heating effect, the addition of a standard solution by tetracycline itself increased the toxicity parameters compared to the raw yolk sample.

The comet test shows the apparent effect of heating on the genotoxicity criterion; heating resulted in a marked increase in comet length, % DNA in tail, and tail moment parameters by 60%, 3000%, and 5000%, respectively. A similar trend was found for samples for which a standard solution of tetracycline was added; comet length, % DNA, and tail moment parameters were increased 96%, 1100%, and 3000%, respectively. Nevertheless, the heating effect, the addition of a standard solution by of tetracycline itself increased the toxicity parameters compared to the raw yolk sample.

## Discussion

In this present study, tetracycline residue was detected in all of the examined samples testing, while confirming a safe status in terms of the levels measured. Indeed, the most contaminated samples contained residual levels at least 30 times lower than the maximum residue level set by EFSA (200ng/g). During the last two decades, several studies have reported on the excessive application of tetracycline in African counties. In Kaduna State, Nigeria, 10 farms were found to use oxytetracycline in egg production in an excessive manner (3). Another study from Enugu state, Nigeria showed that in 25 farms tested, all samples (100%) contained oxytetracycline and concluded that it was the most widely used group of antimicrobial drugs in poultry farms (16). Samples collected from different areas of AL-Qassim in Saudi Arabia showed an average concentration of 145ng/g tetracycline residue, which is higher than that was found in this present study (5). By contrast, there are some examples of very low or negligible concentrations of tetracycline in the literature. Just 2.5% of 320 commercially produced eggs were identified with a measurable amount of tetracycline residue. More than 99% of examined egg samples in Canada were found to be free from any drug residues (17). Similarly, in another study conducted in Japan, no tetracycline was detected in tables eggs sampled from the market (18).

In Iran, there has been a state monitoring program that was implemented under the supervision of the veterinary administration of Iran in the last two decades and all animal-origin products, including eggs, are regularly examined for antibacterial residues nationwide. However, there has been no official interest in releasing the obtained results. Interestingly, we did not find any publication on the safety of eggs in the market with regard to tetracycline concentration in Iran from an independent organization among the national or international scientific literature.

In our present study, no correlation was found between the residue levels and weight of the whole egg, crust, yolk, or albumin of the relevant samples. Commercially produced eggs contained a higher concentration of tetracycline residues than the ones produced locally in homes. Regarding the accumulation of tetracycline in yolk, our results confirmed those of Hsieh and Lian et al who reported that in hens assigned to equal groups (n=20) and fed either 50 or 200 g/ton oxytetracycline for 5 d, residue occurred in yolk (117ng/g) that was two times higher than that in albumen (258 ng/g) (19).

Tetracycline partitioning of the albumin and yolk has been linked to the pharmacokinetics and physiology of chicken and egg formation. Following drug administration, tetracycline residue appears more rapidly in egg albumin than in yolk, but because more lysozyme is degraded in the albumen, the levels are higher in the yolk (20). Thermal liability of tetracycline, as found in the present study, is well characterized. In one study, 8 min boiling of contaminated eggs resulted in a 29.8% reduction in doxycycline concentration (21).

The thermal resistance of chlortetracycline residue was investigated in eggs and showed that after boiling for 15 min, the concentration of chlortetracycline in the yolk decreased by 61% (22). Compared with our present results, the more destructive effect of heat in the latter study can be attributed to the higher primary concentration of tetracycline in the yolk and longer heat treatment. In another study, thermal sanitization of milk resulted in a 5.74%–15.3% reduction in chlortetracycline residues (13). Similarly, another study reported on the destructive effect of boiling on chlortetracycline residue in chicken meat and showed a more marked84%reduction (23). Similar results have been reported for the effect of cooking temperature on tetracycline residue in catfish fillets (24).

The degradation and inactivation of antibiotic residues during heat treatment can be influenced by various factors, including the food matrix temperature and chemical structure of an antibiotic (13).

Generally, the thermal destruction of various veterinary drug residues in food implies that heating may decrease the potential toxic effects of these compounds on consumers (13, 25). Accordingly, severe heat treatment, like cooking, can lead to complete destruction of residues and might have a positive effect by eliminating most heat-liable antibiotic residues in food, like TC and OTC. However, it remains possible that some heat-treated compounds may develop more toxic properties.

In the present study, for the first time, the toxicity of tetracycline residues in raw and heated-treated yolk samples on hepatic cells was investigated by comet assay and our results clearly showed that the thermal processing of egg yolk containing tetracycline residues leads to DNA damage, which causes genotoxicity. Additionally, even if the residues are well below the regulated limits, DNA damage can be observed at the cellular level. This finding is in accordance with the findings of a study that showed heating leaves some degraded compounds of TC in the form of inactive or toxic impurities.

The fate of oxytetracycline during the thermal treatment of chicken and pig meat was studied and further assessed the toxic effects of degraded products on rats. Male rats received an oral daily dose of 10 mg/kg bw OTC thermal derivatives, such as 4-epioxytetracycline (EOTC), a-APO OTC, and B-APO, for 90 d. B-APO OTC treatment could damage the liver and induce necrosis in hepatocytes, but a-APO OTC was not toxic (26, 27).

The toxic effect of 14 antibiotic residues, including tetracycline, in heat-treated samples, was inferred using the Ames test. For tetracycline residue, their results indicated increased mutagenicity in heat-treated samples based on the new peaks formed in the HPLC chromatograms of specimens, which supposedly represented new toxic epimers of tetracycline (28).

## Conclusion

Tetracycline residue highly prevalent, but the extremely low concentration in table eggs sold in a representative Iranian market suggests that tetracycline is used at appropriate concentrations in chicken houses. Although boiling results in the reduction of the measurable tetracycline residue levels, markers in hepatic cells that were generated in the presence of the egg yolks that were tested, genotoxicity tends to increase after the application of heat.

## Ethical consideration

Ethical issues (including plagiarism, informed consent, misconduct, data fabrication and/or falsification, double publication and/or submission, redundancy, etc.) have been completely observed by the authors.
